# Personal

**Published:** 1867-11

**Authors:** 


### PERSONAL.

WE have somewhat recovered from an attack of modesty which we had about a year ago, and from henceforth shall not hesitate to append T. to our editorials when we feel like it, except those that have W. We have come to the conclusion that as we have but few letters to our name, and no title worth speaking of, that we must make the best possible use of them; however we will use but one at a time, and they will hold out longer.

